# The Detection of Foreign Items in Laundry Industry by Dual-Energy X-ray Transmission—Advantages and Limits †

**DOI:** 10.3390/s22218248

**Published:** 2022-10-27

**Authors:** Christine Bauer, Rebecca Wagner, Johannes Leisner

**Affiliations:** Division Development Center X-ray Technology, Fraunhofer Institute for Integrated Circuits IIS, 90768 Fürth, Germany

**Keywords:** X-ray transmission, dual energy, dual-energy X-ray transmission, laundry industry, laundry, basis material decomposition, sorting, laundry processing, sharp injuries

## Abstract

Firefighters, paramedics, nursing staff, and other occupational groups are in constant need of fast and proper cleaning of their professional workwear, not only during a pandemic. Thus, laundry technology needs to become more efficient and automated. Unfortunately, some steps of the cleaning process, such as finding and removing foreign items from pockets or belts, are still completed manually. This is not just time-consuming but potentially dangerous for the workers due to the hazardous nature of items such as scissors, scalpels, or syringes. Additionally, some items may damage the garments by staining or harm the laundry machines, causing malfunctions and process failure. On the one hand, these foreign items are often hidden inside the clothes, making detection very challenging with conventional superficial sensors. On the other hand, these items can be diverse and cannot be detected by metal detectors alone. X-ray transmission has proven to be a powerful tool for detecting items inside of objects. The dual-energy approach (DE-XRT) even allows obtaining quantitative information about the chemical composition of the measured materials. In this study, working garments were accompanied and filled with realistic foreign items. The potential of DE-XRT to detect those items was successfully shown.

## 1. Introduction

In a global pandemic, meticulous hygiene and careful precautions are crucial. These are not only important while working and acting with patients but also in other areas of everyday life. Nursing and medical staff require properly cleaned workwear, uniforms, and linen, not only during a pandemic [1]. Laundry personnel are processing high amounts of contaminated linen every year and are thus at risk of injury by physical (e.g., noise and temperature), chemical (bleach, detergents, soaps), ergonomic (heavy lifting and carrying), and biological (body fluids of all kinds) hazards. A study showed that 92.5% of hospital laundry workers were in contact with contaminated linen and 67.9% were exposed to sharp objects [2]. In the healthcare laundry environment, exposure to bloodborne pathogens such as hepatitis B, hepatitis A, or the human immunodeficiency virus HIV [3,4,5] happens when contaminated garments, handled by the laundry laborers, contain or are accompanied by sharp elements [6], mostly due to a lack of attention from the medical personnel [7]. Sharp items are, as defined by the Occupational Safety and Health Administration (USA) [8], objects which can penetrate the skin, such as needles, scalpels, or pieces of broken glass [9], as well as teeth or bone splinters [10]. It was shown that sharps contamination can be reduced to some extent with staff education, but cannot be excluded completely [11]. Besides sharps, expensive medical equipment finds its way into soiled garment and is thus lost for further use [12].

Industrial laundry is not only used in the hospital environment. Firefighters, paramedics, civil protection organizations, and the military, among others, depend on laundry services. Here, the bandwidth of items that can be found in pockets, belts, sleeping bags, or uniforms is even bigger. Besides medical items, personal belongings such as mobile phones, paper, and lipstick may find their way into the laundry. Those items may be non-hazardous for the laundry staff but harmful to the machines or garments themselves. Special cases are pens, highlighters, and permanent markers, which may stain the linen and laundry itself, resulting in costs of about EUR 1500 per undetected pen for rewashing and replacing the damaged garment [13].

Only some of the aforementioned objects can be found by metal detectors. One approach to detect foreign items in laundry garments and linen is to use a holographic ultrasonic device [14]. Another method, which is already used for finding foreign bodies in some highly automated industrial laundry processing lines, is X-ray transmission (XRT) [15]. XRT is able to find thick metallic objects in laundry, but will fail to detect organic materials such as plastics and thin metal pieces, especially aluminum. An advanced version of XRT, which may be a powerful tool for detecting foreign items in workwear and linen, and thus prevent sharp injuries and damages, is dual-energy X-ray transmission (DE-XRT). The analysis of DE-XRT images yields material information on the scanned objects in real time, making an integration into online processes possible. DE-XRT is mostly used for non-destructive testing and in the security sector, e.g., in luggage scanners at airports, but also shows promising results in mining [16,17,18], food safety [19,20], recycling [21,22,23,24], and other sorting applications [1,25,26,27,28]. As DE-XRT is able to distinguish different compounds or elements, it is well suited for finding a variety of hazardous and non-hazardous materials in laundry, such as plastics, paper, metal, and glass. This property of DE-XRT outclasses the standard XRT. An integration of DE-XRT at one of the conveyor belts of a laundry processing line could thus prove advantageous and help to protect laundry workers, as well as clothes and machines.

This paper provides a short introduction to the method of DE-XRT. It then presents results on the examination of paramedical workwear filled with real-life foreign items of a wide range of materials using DE-XRT. These proof-of-principle experiments carried out in a laboratory setup show the advantages and limitations of the approach. Further, some points concerning a possible future integration of DE-XRT into a laundry processing line are discussed.

## 2. Materials and Methods

### 2.1. Samples

**Figure 1:**
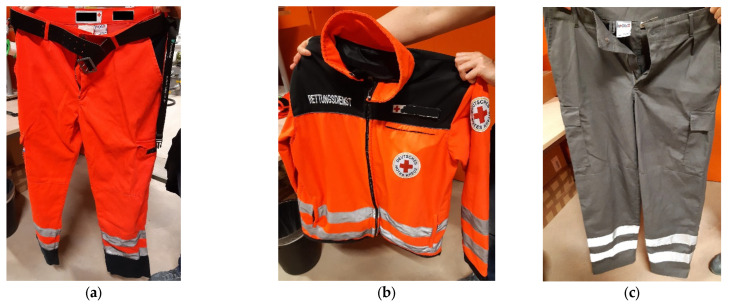
Photos of the investigated workwear. (**a**) Paramedical trousers; (**b**) paramedical jacket; (**c**) civil protection trousers.

In this study, three samples were examined, representing typical workwear that is used by paramedical staff in Germany. The following garments were used:

1. a pair of paramedical trousers with a belt and reflector stripes (Figure 1a);
2. a paramedical jacket with reflector stripes, metallic zippers, and press studs (Figure 1b);
3. a pair of civil protection trousers with reflector stripes, metallic zippers, and a trouser button (Figure 1c).

Linen and hospital workwear is typically thinner and does not contain the variety of materials present in the selected samples. It can therefore be assumed that the chosen approach will work even better for hospital laundry. Therefore, it is not part of this study.

For the foreign items, objects were chosen, which

1. may stain clothes, such as ball pens, marker pens, fine liners, lipsticks, and lip balms;
2. harm the laundry machines, such as mobile phones, batteries, foldback and paper clips, paper notepads, tapes, and carabiners;
3. are dangerous for laundry staff, such as safety scissors, utility knives, and screwdrivers.

The items described above consist of different materials, namely steel, aluminum, a variety of plastics, and other organic components (e.g., paper or wax).

### 2.2. Dual-Energy X-ray Transmission

**Figure 2:**
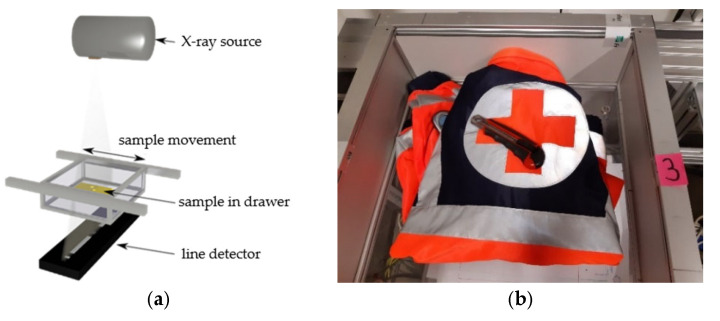
(**a**) Schematic of the used drawer system equipped with a dual-energy line detector and an X-ray source (adapted from [26], licensed under CC BY 4.0). (**b**) Example of workwear placed in the drawer system and loaded with a cutter, a mobile phone, and other foreign items.

To obtain the DE-XRT images, a laboratory set-up with a drawer system was used (Figure 2a), where the sample was transported between an X-ray source (Comet MXR-225HP/11) and the dual-energy line detector (Hamamatsu C10800-09FCM-C). The detector had a pixel pitch of 0.4 mm and consisted of two sensor layers. With this set-up, it was possible to record two images at low and high energy *E* (LE and HE, respectively), simultaneously. The measurements were performed with 100 kV, 5 mA, and an exposure time of 2.67 ms per recorded line. The drawer with the sample inside was moved with a speed of approximately 170 mm/s (Figure 2b).

Subsequently, a method called basis material decomposition (BMD) was used to analyze the two dual-energy images (LE and HE) obtained for each measurement. This method, which can analyze images in real time, is described in detail in (open-access) publications [19,26,29,30]. In short, BMD is based on the Lambert–Beer law

(1) $$I\left(E\right)=I_{0}\left(E\right)\exp\left(-\underset{i}{\sum }{\mu^{\prime }}_{i}\left(E\right)p_{i}\right),$$

which gives the X-ray intensity *I* behind an object. Essentially, this intensity depends on the X-ray spectrum *I*_0_ used to illuminate a sample and on the X-ray mass attenuation coefficients *μ*′ as well as the areal densities *p* (i.e., the mass per area) of all materials *i* through which the X-rays pass. While X-ray spectra can be determined by a careful characterization of the X-ray facility [31], mass attenuation coefficients *μ*′ can be found in tables for chemical elements [32]. For other materials, *μ*′ can be calculated based on the weight fractions $w_{j}$ of the chemical elements that they are made of using the following formula [32]:

(2) $$\mu^{\prime }=\underset{j}{\sum }w_{j}{\mu^{\prime }}_{j}$$

Therefore, only the areal densities are unknown. The measurement of the transmitted intensity with two different known X-ray spectra can be described by two equations in the form of Equation (1). This equation system can be solved to obtain the areal densities of two chosen basis materials.

**Figure 3:**
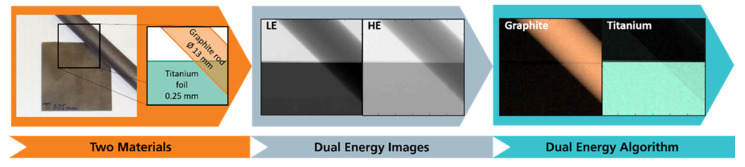
Schematic of the data-processing steps of the basis material decomposition, for simplification with two pure materials: a graphite rod partly overlapping a titanium foil. LE and HE indicate the two images (low and high energy) obtained by the dual-energy X-ray detector which do not allow a differentiation of the two materials. The analysis yields two basis material images showing only graphite or titanium, respectively (created from data published previously in [29]).

If a sample consists of only two materials, as in the example presented in Figure 3, it is appropriate to choose those as basis materials. In this case, the two resulting basis material images show the areal density of the respective basis material for each pixel. Thus, it is possible to distinguish the two materials even if they overlap. If further materials are present, they appear as a superposition of the chosen basis materials [26].

For the presented application, one of the basis materials had to represent the clothes. However, their chemical composition was not known in detail. Therefore, their X-ray mass attenuation coefficient could not be calculated using Equation (2). Yet, threads are made mainly of C (atomic number *Z* = 6), O (*Z* = 8), H (*Z* = 1), and sometimes N (*Z* = 7). For instance, the main component of cotton is cellulose with the chemical formula (C_12_H_20_O_10_)_n_. This knowledge was used to obtain an initial guess for the X-ray attenuation of a suitable basis material. From the chemical composition, an effective atomic number can be calculated using

(3) $$Z_{\mathrm{eff}}^{k}=\frac{\sum_{j}Z_{j}^{k}\rho_{j}}{\sum_{j}\rho_{j}},\;k\approx 3,$$

with the partial chemical densities *ρ*. For cellulose, this gives an effective atomic number *Z*_eff_ ≈ 6.9. Its X-ray attenuation can thus be approximated by a weighted average of the attenuations of C and N. By varying *Z*_eff_ slightly, it was attempted to obtain basis material images, where the clothes are visible in one image only, while the other basis material image shows foreign items.

## 3. Results

**Figure 4:**
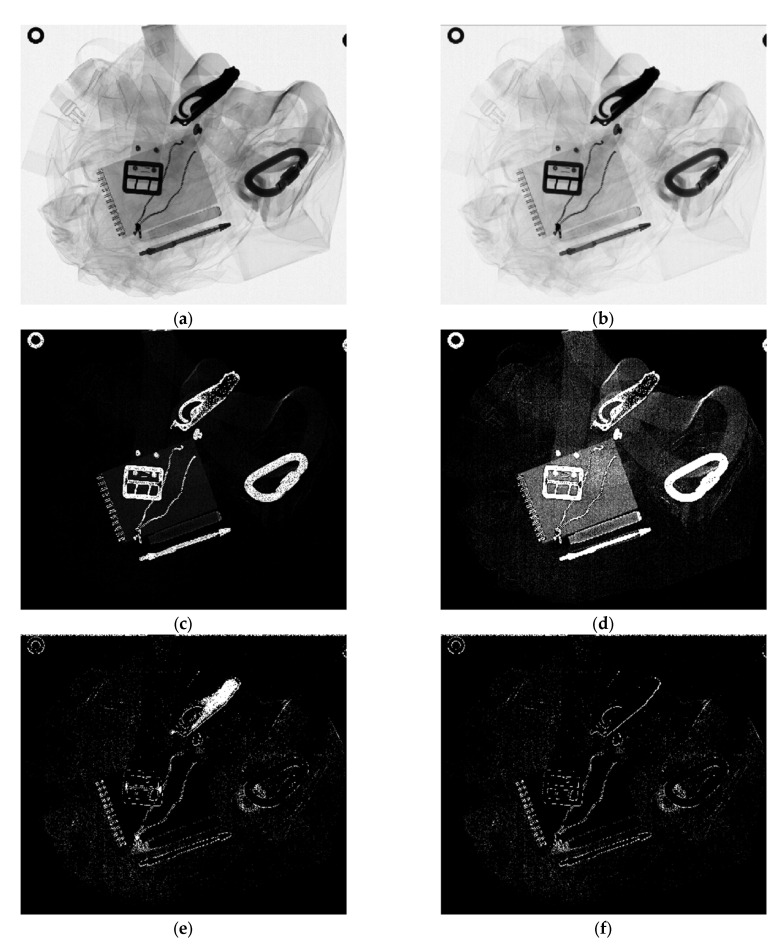
(**a**) Low-energy image of the paramedical trousers (adapted from [26], licensed under CC BY 4.0); (**b**) high-energy image; (**c**) areal density image with *Z*_eff_ = 26; (**d**) areal density image with *Z*_eff_ = 11.2; (**e**) areal density image with *Z*_eff_ = 6; (**f**) areal density image with *Z*_eff_ = 6 after removing areas of low X-ray transmission. As the second basis material, *Z*_eff_ = 7.1 was used in all cases.

Figure 4a,b show the LE and HE XRT images of paramedical trousers, respectively. While metal parts are clearly visible due to their high absorption, items such as a belt, notepad, and marker pen cannot be distinguished from thicker parts of the clothes based on their gray values.

The aim of BMD is to separate clothes and foreign items into separate images. Therefore, one of the basis materials is chosen to represent the clothing. An effective atomic number *Z*_eff_ = 7.1 is used to describe its X-ray attenuation, which is slightly higher than that of nitrogen with *Z* = 7. The second basis material has to be chosen so as to match the foreign items. Due to their variety, different options exist. As mentioned in Section 2.2, objects not made of one of the two chosen basis materials appear as a superposition of them. Therefore, the choice of the second basis material also influences the image of the first basis material.

Figure 4c shows a basis material image with *Z* = 26, that is, all the parts made of iron and similar materials, such as a carabiner, a belt buckle, parts of the safety scissors, and a pen refill. All other foreign objects have only a small signal in this image and appear mainly in the basis material image of the cloth to which their *Z*_eff_ is closer (not shown).

Figure 4d shows a basis material image with *Z*_eff_ = 11.2 as the second basis material. Here, a belt, a paper notepad and a marker pen are visible. As *Z*_eff_ = 11.2 is an appropriate choice for these items, they do not show up in the basis material image showing the clothes (not shown). However, the iron-like objects found in Figure 4c can also be seen in Figure 4d. As these are not made of one of the basis materials used here, their high signal in Figure 4d is compensated by a negative signal in the basis material image of the cloth (not shown). This enables the removal of these objects from the basis material image of the foreign items.

Figure 4e shows the basis material image if *Z* = 6 is chosen for the second basis material. This image shows the cap of the marker pen and a roll of tape. All other foreign items have higher atomic numbers and thus do not appear in this image, but in the image showing the clothes with the higher *Z*_eff_ = 7.1 (an exception is discussed below). Figure 4e is noisier than the images shown before due to the small difference in *Z*_eff_ between the two basis materials. It also shows the outlines of some objects made of materials with higher *Z*_eff_, such as the belt buckle or carabiner. These are artifacts created when pixels are incompletely covered by objects. They could, in principle, be removed by erosion of the BMD image. This was not carried out for two reasons. First, erosion would also remove fine structures such as needles, which are a danger to laundry workers. Second, the artefacts do not hinder the interpretation of larger structures.

**Figure 5:**
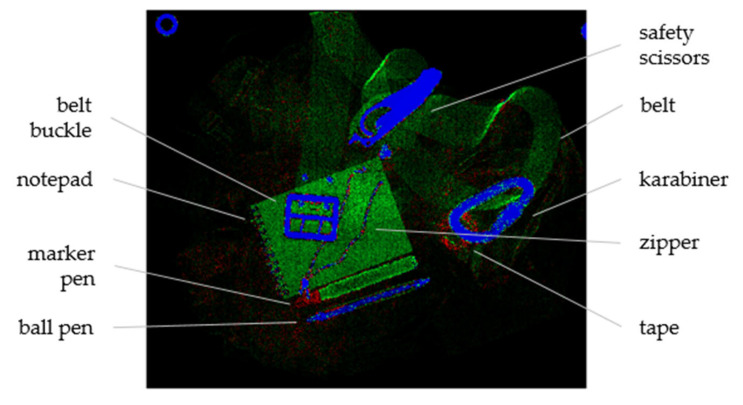
False-colored combination of the material images shown in Figure 4; red indicates *Z*_eff_ = 6 (plastics), green indicates *Z*_eff_ = 11.2, and blue indicates *Z*_eff_ = 26 (iron). Adapted from [26], licensed under CC BY 4.0.

The three basis material images with the corrections discussed above can be used as the blue, green, and red channels of a false-color image (Figure 5). One further adaption is performed to the three-color channels. Figure 4e also shows parts of the safety scissors, which indicates that they are made of plastic. However, this is not true. Their appearance in this image is due to the fact that no signal is detected behind this thick metal part because of its high X-ray attenuation. Consequently, the BMD analysis fails. However, the low detected intensity can be used to identify this part as metal. Thus, areas with too low transmitted intensity are removed from the red (Figure 4f) and green channels and are colored blue instead. The false-color representation (Figure 5) permits the visualization of all foreign objects in one image that also includes material information. This allows their detection and subsequent removal.

**Figure 6:**
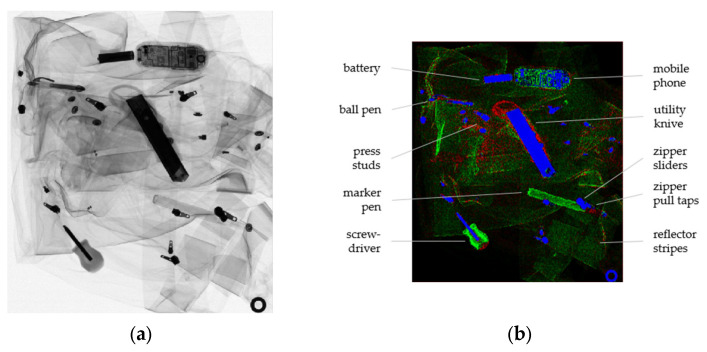
(**a**) High-energy image of the paramedical jacket. (**b**) False-colored combination of material images; red indicates *Z*_eff_ = 6 (plastics), green indicates *Z*_eff_ = 11 (sodium), and blue indicates *Z*_eff_ = 26 (iron). As the second basis material, *Z*_eff_ = 7.1 was used in all cases.

In the same way as illustrated with Figure 4 and Figure 5, the false-color image of a paramedical jacket in Figure 6 is created. Here, the green channel also shows the reflector stripes and the jacket lining, while the blue channel shows press studs, as well as sliders and pull tabs of zippers. These are not foreign items, but parts of the clothes. However, they appear in the false-color image as they are made of materials different from the remaining cloth but similar to the foreign items. Apart from these components of the jacket, a battery, a mobile phone, a utility knife, a marker pen, and a screwdriver are visible. Some of these items may not only harm the machine but also laundry workers. In particular, the marker pen is hard to distinguish from the clothes in the XRT image (Figure 6a) without BMD analysis.

**Figure 7:**
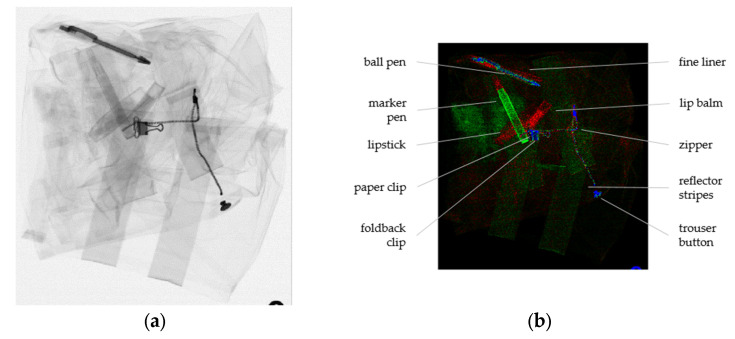
(**a**) High-energy image of the civil protection trousers; (**b**) False-colored combination of material images, red indicates *Z*_eff_ = 6 (plastics), green *Z*_eff_ = 13 (aluminum) and blue *Z*_eff_ = 20 (calcium). As the second basis material, *Z*_eff_ = 7.3 was used in all cases.

Another example is shown in Figure 7. Here too, the green and blue channels show parts of the garment: the reflector stripes, a trouser button, and a zipper. Further, a few foreign items that may stain the trousers during the washing process can be recognized: several kinds of pens and a lipstick. Again, these can easily be missed in a simple XRT image (Figure 7a), but are clearly visible after analysis of the DE-XRT images by BMD (Figure 7b).

## 4. Discussion

The examples presented above show that DE-XRT can visualize foreign items in laundry, some of which would be hard to detect by standard XRT. Detectable materials range from organics to metals. This versatility allows visualizing objects that may stain the clothes (pens, lipsticks), damage the machine (batteries), are at risk themselves (objects of value such as mobile phones, paper notepads), or impose a risk of injury to laundry workers (knives, scissors).

Not only can foreign objects be detected, but in many cases they can even be recognized after gaining some practice in interpreting the kind of images delivered by the presented method. This makes removing the objectionable items safer for laundry workers, as they can judge when special care is needed to avoid injuring themselves. DE-XRT can thus contribute to an improvement in occupational safety and reduce incapacitation of staff due to injuries.

For garments made of only one type of cloth, or at least types with very similar *Z*_eff_, a neat separation of cloth and foreign items is possible. For more complex clothes, for instance with reflector stripes or metal parts, such as press studs and zippers, some parts may show up in one image with the foreign items. Still, with some experience, laundry personnel may be able to distinguish these parts from harmful objects. Thus, unnecessary searching of these pieces of laundry would be avoided.

A challenge to the method is posed by items with *Z*_eff_ close to that of the clothes themselves. Typically, this means organic substances such as plastics, lipsticks, or food. The small contrast in *Z*_eff_ leads to noisy BMD results, which makes interpretation more difficult. The red channel of the false-color images thus requires more attention among laundry workers.

The situation is further complicated as *Z*_eff_ is not identical for different clothes. For instance, a slightly higher value is used for the civil protection trousers (Figure 6) compared to the other examples. While such an optimization is possible on a laboratory scale, an adaption of *Z*_eff_ for every piece of garment is not realistic in a working environment. For facilities specializing in hospital laundry, where all clothes are made of similar materials, this should not even be necessary. For facilities that have to handle a wider range of cloth materials, a representative selection of garments would be measured during implementation of the system to determine an average *Z*_eff_. This compromise would degrade the BMD result for basis materials with small contrast in *Z*_eff_. Thus, traces of cloth may be visible in the same basis material image as foreign items of organic materials, i.e., the red channel in the false-color images. However, until now, experience shows only small variations in *Z*_eff_ for different garments. Thus, the effect on the green and blue channels, that would typically show materials with *Z*_eff_ similar to Al and Fe, respectively, can be neglected.

Currently, all pockets have to be searched manually, which limits the speed at which clothes can be moved through the laundry machine. DE-XRT reduces the number of pieces that have to be processed manually to those that really contain foreign items. This can increase the throughput of the facility. The BMD analysis does not limit the speed, as it works in real time. However, a limit is given by the time needed by laundry workers to interpret the images and decide whether objects have to be removed from, or are an inherent part of, a piece of laundry.

This task could be assisted by machine learning algorithms. These require a large amount of training data that are difficult to obtain with a laboratory scale set-up. Therefore, industrial project partners are sought for further development and automation of the concept. One approach would be the use of BMD images, where parts such as zippers, press studs, or buttons are labelled, as training data for the algorithms. These could help to recognize the aforementioned parts and reduce their intensity in the images, thus drawing more attention to the foreign items. Similarly, the algorithms could be further trained to identify items that pose a risk of injury by providing BMD images with the corresponding labels. Then, it would be possible to highlight these as a warning to the people who have to remove them from the piece of clothing by hand. This may pave the way to Internet of Things (IoT)-based laundry services, which combine Big Data analytics with intelligent logistics management and machine learning techniques [33].

Another point to consider is that professional laundry service is a resource-intensive process, which uses large amounts of water, detergents, and electrical energy [34]. Using DE-XRT during the process may avoid the need to repeat washing and thus reduce the amount of energy, waste water, and detergent, while prolonging the lifespan of clothing and laundry machines. Besides a reduction in the environmental impact, this helps to reduce costs.

Certainly, it needs to be ensured that these improvements are not negated by the use of expensive and energy-intensive X-ray technology itself. Moreover, the need for radiation protection and training of personnel has to be taken into account. To obtain a rough estimate of the training time, hand luggage and freight inspection at airports can be considered as a reference, because similar images have to be judged. The training of this personnel takes approximately three to twelve weeks [35,36]. However, the interpretation of X-ray images is only a small part of it. Most other parts, such as the legal basis, security checks on the passengers, or knowledge on weapons and explosives, are not needed in laundry facilities. Thus, the training needed to detect foreign items in clothes should be possible within a few days.

The advantages and costs may have to be judged in all individual cases. However, laundry machines are already on the market that use ordinary X-ray transmission to visualize objects with stronger X-ray attenuation [13]. In this case, the investment would mainly consist of replacing the detector, buying new software, and training the personnel. As a consequence, training should not take long as the workers are already used to X-ray images and thus only need to learn interpretation of the additional information provided by DE-XRT. The advantage of this method would be material sensitivity that allows to visualize also materials of lower atomic number and thinner metal pieces. The obtained material information would also help to identify objects more easily.

## Data Availability

Not applicable.
